# Phylogenetic Analysis of Rare and Endangered *Tulipa* Species (*Liliaceae*) of Kazakhstan Based on Universal Barcoding Markers

**DOI:** 10.3390/biology13060365

**Published:** 2024-05-22

**Authors:** Maxim Sutula, Ayan Kakanay, Dilnur Tussipkan, Samatulla Dzhumanov, Shuga Manabayeva

**Affiliations:** 1 National Center for Biotechnology, Astana 010000, Kazakhstan; m.sutula@biocenter.kz (M.S.); kakanay@biocenter.kz (A.K.); tussipkan@biocenter.kz (D.T.); 2 Aksu-Zhabagly State Nature Reserve, Zhabagly 161310, Kazakhstan; samat.reserve@mail.ru; 3 Faculty of Natural Sciences, L.N. Gumilyov Eurasian National University, Astana 010008, Kazakhstan

**Keywords:** *Tulipa*, DNA barcoding, hybridization, ITS, *mat*K, phylogenetics and systematics, *rbc*L

## Abstract

**Simple Summary:**

In Kazakhstan, tulips are not just beautiful flowers but also vital to the environment, with 35 species, 18 of which are protected. Recent research near Kazakhstan’s borders uncovered new tulip varieties, highlighting the need for a species inventory. In this study, we identified eight tulip species using both physical traits and advanced DNA testing. By analyzing genetic markers, we discovered that certain DNA regions are particularly useful for identifying tulip species. However, when it comes to closely related tulips, combining data from multiple DNA markers is crucial for accurate classification. This study also unveiled possible natural hybrids, indicating complex interactions between different tulip species. This research sheds light on the diversity and evolutionary relationships among tulips, providing valuable insights for conservation efforts and understanding plant genetics. Further investigations into tulip populations are necessary to confirm these findings and better protect these precious flowers.

**Abstract:**

In Kazakhstan, the genus *Tulipa* is represented by 35 species, 18 of which are listed in the Red Data Book of Kazakhstan and protected by the state. Recent studies of tulip specimens from regions bordering Kazakhstan emphasize the significance of species inventory and report the discovery of several hybrids. In this study, eight tulip species were identified based on morphological characteristics and using DNA barcoding methods. Molecular genetic markers, including nrDNA (ITS) and cpDNA markers (*rbc*L, *mat*K), of the studied species were sequenced and analyzed using the Bayesian inference and maximum likelihood phylogenetic analysis methods. Our work demonstrates that DNA barcodes based on the ITS, *rbc*L, and *mat*K marker regions have successful practical applicability, with ITS being the most informative at the intragenic level. However, for distinguishing closely related taxa, the most effective approach would be to use a combined dataset of sequences from multiple DNA markers. The results showed discrepancies in the placement of several taxa (*T. kaufmanniana*, *T. patens*), likely due to introgression and natural spontaneous hybridization. The molecular phylogenetic analysis suggests the existence of a previously undescribed hybrid between *T. patens* and *T. alberti*. Further detailed population studies are needed to validate this hypothesis.

## 1. Introduction

The genus *Tulipa* L. (*Liliaceae* family, *Lilieae* tribe) contains about 75 species and is divided into four subgenera: *Clusianae* (Baker) Zonn. (4 species), *Orithyia* (D. Don) Baker (4 species), *Eriostemones* Raamsd. (16 species) and *Tulipa* Raamsd. (52 species) [1]. Most species have the same basic number of chromosomes (2n = 2x = 24) [2]. Tulips grow and develop well at 22–30 °C, but can survive at 37–42 °C with sufficient humidity. Tulip is a spring-blooming perennial that flowers from March to May [3]. Tulip flowers are bulbiferous and contain five major parts: the basal plate, basal stem, fleshy scales, flower bud, and tunic. The flowers have a wide variety of colors with different components, generally pigmented red, pink, yellow, or white [3]. Kazakhstan’s endemic and rare tulips thrive in a variety of landscapes and altitudes. *Tulipa greigii* (Regel), found in valleys and foothills up to 2400 m high, blooms from April to June with cup-shaped flowers that are red, orange, or cream, sometimes with a black or yellow base. *Tulipa patens* (C. Agardh ex Schult. and Schult. f.), common in northeastern regions, blooms from mid-April to May with white or pink flowers with a distinctive yellow spot. *Tulipa bifloriformis* (Vved.), native to the Tian Shan Mountains, blooms from March to May with fragrant flowers that have white perianth leaves with yellow bases. *Tulipa turkestanica* (Regel), found in central and southern areas, blooms from March to June, bearing white flowers with yellow spots, occasionally with purple backs [3,4,5].

Most wild tulips are found in the mountainous regions of Central Asia [4]. Currently, the species is distributed throughout Europe, North Africa, and Asia [5], and is even found in the western Himalayas, southern Siberia, and Inner Mongolia [6]. In fact, Tien Shan Mountain is one of the main diversity centers of *Tulipa* [6]. The genus *Tulipa* is represented by 63 wild species in Central Asia [7], 37 of which are native to Kazakhstan and distributed throughout the country. In total, there are 35 species of wild tulips in Kazakhstan, 18 of which are listed in the Red Data Book of Kazakhstan and are protected by the state [8]. Although there are several endemic species of *Tulipa*, most taxa span the borders of several countries. Since no *Tulipa* taxa from this region are globally red-listed, national-level conservation assessments are an important resource [4]. Tulips are important to the environment because they attract pollinators, support biodiversity by providing a habitat and food, and contribute to soil stability through their root systems.

Our study focused on eight *Tulipa* sp., including *T. greigii*, *T. kaufmanniana* (Regel), *T. turkestanica*, *T. bifloriformis*, *T. patens*, *T. dubia* (Vved.), *T. alberti* (Regel), and *T. schrenkii* (Regel) across the subgenera *Tulipa* and *Eriostemones*. These species are endemic to Kazakhstan, and occur in the vicinity of the cities of Turkestan and Kyzylorda, the western extreme of the Zailiisky, Kungei Alatau, Kyrgyz Alatau, and Chu-Ili Mountains, as well as Karatau and the south of the Betpakdala desert [9]. Their core habitats are loamy steppes, foothills slopes, and hills. They are listed in the Red Book of Kazakhstan as a rare and endangered species [8]. The main limiting factors for their distribution are plowing of land and grazing. However, the threats posed to these tulips are still poorly understood, especially climate change. DNA barcoding plays a key role in the conservation of endangered Tulipa species through several means: species identification and validation, genetic diversity assessment, population monitoring and regulation, detection of hybridization and genetic contamination, forensic analysis for illegal trade, and facilitation of reintroduction initiatives. In essence, DNA barcoding serves as an indispensable tool in the conservation and management of endangered Tulipa species, strengthening efforts to ensure their survival in the face of challenges such as habitat degradation and climate variability. By integrating genetic knowledge with traditional conservation methods, DNA barcoding strengthens our ability to protect these iconic and ecologically important plants for the benefit of future generations [10].

A standardized DNA barcode is a short (<1000 bp) and highly variable segment of DNA derived from specific regions of DNA [11], and can serve as an effective tool for studying biological phenomena. Since 2003, it has been used to identify species, infer ecological and evolutionary relationships between species, and accelerate taxonomic discovery. It has also been used for germplasm conservation, community assembly, species interaction networks, and the assessment of priority areas for environmental protection [12,13]. Overall, progress is being made to apply DNA barcoding for all plant groups and make these data publicly available to assess, conserve, and appropriately use the world’s biodiversity.

Several reviews have highlighted recent barcoding studies in extensive studies of the kingdom Plantae [14,15,16,17]. Four primary gene regions (*rbc*L, *matK*, *trn*H-*psb*A, and ITS) have been generally accepted as the standardized universal DNA barcodes for routine applications in plant species. This provides a platform for the establishment of a centralized plant barcode database [18]. To date, more than 90,000 plant species from around the world have been evaluated using common nuclear and chloroplast markers (ITS, ITS2, *matK*, *rbc*L, *trn*H-*psb*A, *psb*K-*psb*I, *rpo*B, *rpo*C1, *atp*F-*atp*H, *ndh*F, *ycf*1, and *ycf*1b) [17,19,20,21,22,23,24,25,26]. This has led to the recommendation of *rbc*L, *matK*, and ITS as the most suitable markers for broad applications in the regional flora [13,27,28,29].

In the process of marker selection, we compared existing complete chloroplast genomes of *Tulipa* species available in public databases to identify suitable markers [30,31]. The markers used in our study were selected based on their demonstrated utility in phylogenetic studies and their potential for reliable amplification and sequencing. Unfortunately, despite their potential to reveal information on some markers, we encountered challenges in confirming the reliable reproducibility of amplification and sequencing. In particular, we were unable to consistently reproduce amplification and sequencing for markers such as *atp*F-*atp*H, and *psb*K-*psb*I. As a result, we chose the universal DNA markers ITS, *rbc*L, *mat*K, and *psb*A-*trn*H, which were easily amplified and sequenced with 100% reproducibility.

Over the past decade, numerous studies have investigated the genetic diversity of the *Liliaceae* family and the genus *Tulipa*, using various genetic barcoding markers and sequencing techniques [1,5,29,30,31,32,33,34]. The generations and analysis of expressed sequence tags in the extremely large genomes of *Tulipa* were published in 2012. In this study, the first set with 81,791 contigs with an average length of 514 bp was developed for tulip, providing a platform for improving genetic research [35]. Christenhusz et al. investigated the phylogenetic relationships of 25 accessions, representing 23 species in the genus *Tulipa* using DNA sequences from five plastid regions (*trn*L intron and *trn*L-*trn*F spacer, *rpl*16 intron, *rps*12-*rpl*20 intergenic spacer, and *mat*K) and the internal transcribed spacer (ITS) region of nuclear ribosomal DNA [1]. The genetic diversity of *T. edulis* collected from eight different regions in China was studied using four plastid (*rbc*L, *psb*A-*trn*H, *mat*K, and *trn*L-*trn*F) and ITS markers [33]. In total, 15 species of *Tulipa* from Uzbekistan were sequenced and characterized in terms of their phylogenetic relationship using four plastid (*rbc*L, *psb*A-*trn*H, *mat*K, and *trn*L-*trn*F) and ITS markers [5]. Eight taxa including six species and two subspecies of the genus *Tulipa* from Kosovo were investigated using the plastid markers *trn*L-*trn*F, *rbc*L, and *psb*A-*trn*H and ITS markers [36]. All of the above underscore the need to use diverse DNA markers and methods to analyze phylogenetic relationships and population structures, thereby providing valuable information and a platform for the advancement of genetic research.

Our research highlights the importance of genetic analysis in understanding the diversity and distribution of tulips in Kazakhstan. The results of our study not only expand our knowledge of the genetic landscape of tulip species, but also have practical implications for the development of new strategies for monitoring and controlling the movement and distribution of rare and protected tulip species. For example, based on our SNP results, PCR-based tests can be developed to identify tulip species. In addition, we discovered new tulip hybrids, which greatly enhances our understanding of genetic diversity, evolutionary processes, and conservation efforts.

## 2. Results

**Figure 1:**
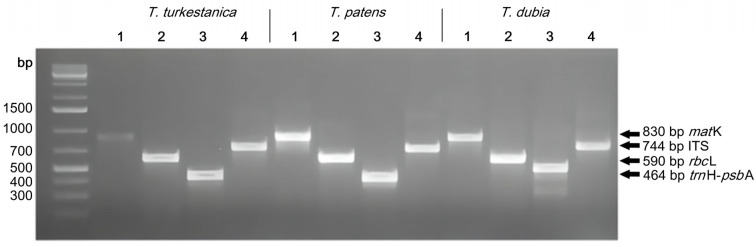
DNA amplification in 2% agarose gel for 1—*mat*K; 2—*rbc*L; 3—*psb*A-*trn*H; 4—ITS.

The agarose gel profile representing the PCR products showed the robust reproducibility of the amplification and sequencing results across all DNA markers used (Figure 1). To ensure accurate sequencing, the beginning and end of the sequences were trimmed. Amplicon sizes were as follows: *rbc*L varied from 564 to 590 bp, *trn*H-*psb*A varied from 464 to 528 bp, *mat*K varied from 598 to 865 bp, and ITS varied from 607 to 744 bp. Statistical errors were minimized by performing at least three technical and biological replicates of each PCR reaction. Figure 1 shows one of these replicates.

Amplification of DNA barcodes was conducted with high efficiency ranging from 91% to 99%. Sequences of Kazakhstan *Tulipa* sp. were submitted to the NCBI database with the accession numbers given in Table 1.

The basic indicators of genetic diversity were examined, including nucleotide divergence (Pi), and the proportion of conservative (C), polymorphic, and segregating (S) regions (Table 2). The ITS regions showed the highest divergence (Pi = 0.05), with the proportion of conservative regions reaching 81.8%, while the proportion of polymorphic regions varied around 18.2%. Conversely, the *rbc*L regions showed higher conservatism (Pi = 0.002), with 97.8% conservative regions and only 2.2% variable regions. The *mat*K regions were characterized by intermediate values (Pi = 0.007), with 96% conservative regions and 4% variable regions. The G + C content of the aligned sequence of the analyzed markers varied between 34.6 and 60.0%.

The use of the BLAST tool to search for identical sequences within the NCBI database revealed limited effectiveness at the species level for the chloroplast DNA markers rbcL and *mat*K. The search within the NCBI database using the ITS DNA marker successfully identified only the species *T. greigii*, *T. kaufmanniana*, *T. bifloriformis*, and *T. alberti* with 100% accuracy. However, intra-specific discrepancies were observed for other species: *T. turkestanica* (99.79% similarity with *T. bifloriformis*), *T. patens* (100% similarity with *T. alberti*, 99.79% with *T. kaufmanniana*), *T. dubia* (100% similarity with *T. turkestanica*, 99.37% with *T. bifloriformis*), and *T. schrenkii* (100% similarity with *T. turkestanica*, 99.37% with *T. bifloriformis*). Nevertheless, all DNA barcodes investigated successfully identified species at the genus level with 100% accuracy.

### 2.1. Single-Nucleotide Polymorphisms of the ITS, rbcL, and matK DNA Sequences in Tulipa

All marker genes were successfully sequenced with 100% accuracy. The DNA sequences of ITS regions in tulips showed considerable diversity. The sequences of *T. turkestanica* and *T. bifloriformis* showed similarity but not complete identity. Only one *T. greigii-*specific single-nucleotide polymorphism (SNP) at position 34 and one *T. bifloriformis-*specific SNP at position 339 were detected (Table 3).

The intraspecific sequence variation among *Tulipa* sp. ranged from 0 to 0.29%, with a mean interspecific distance of 0.03%. The cpDNA sequences of the *rbc*L and *mat*K regions showed a high degree of conservation. The sequences of the studied species were found to be identical. Informative sites with high variability were detected only at two positions after alignment: *rbc*L—117, 212; *mat*K—174, 302. A single-nucleotide polymorphism (SNP) specific to *T. bifloriformis* was identified at positions *rbc*L, 255; *mat*K, 314 (Table 3). Among the representatives of the genus *Tulipa*, an extremely low number of sequence differences were observed (ranging from 0 to 0.06), with a mean interspecific distance of 0.02%.

### 2.2. Nuclear rDNA Phylogeny

**Figure 2:**
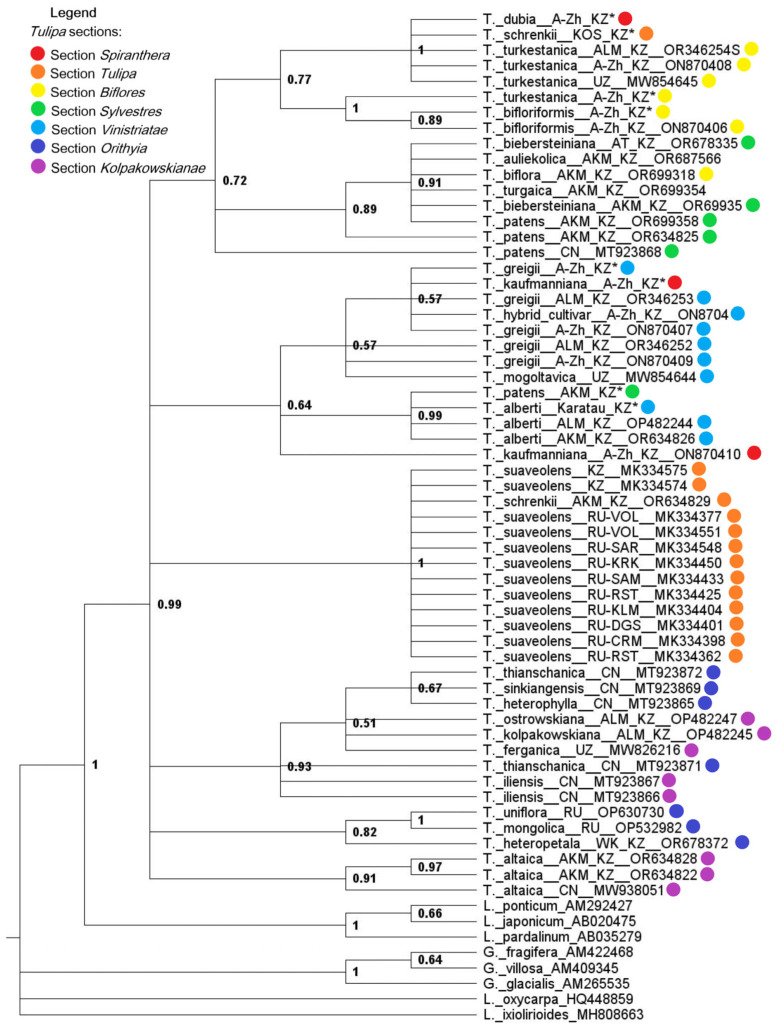
Bayesian inference 50% majority rule consensus tree based on ITS sequences, including posterior probabilities (PP > 0.5) provided above each branch. The locations of *Tulipa* samples retrieved from the NCBI GenBank are indicated by capital letters representing the following: Kazakhstan (KZ), China (CN), Russia (RU), and Uzbekistan (UZ). Tulip samples investigated in this study are marked with an asterisk (*). *Tulipa* sections are represented by different colors on the tree.

**Figure 3:**
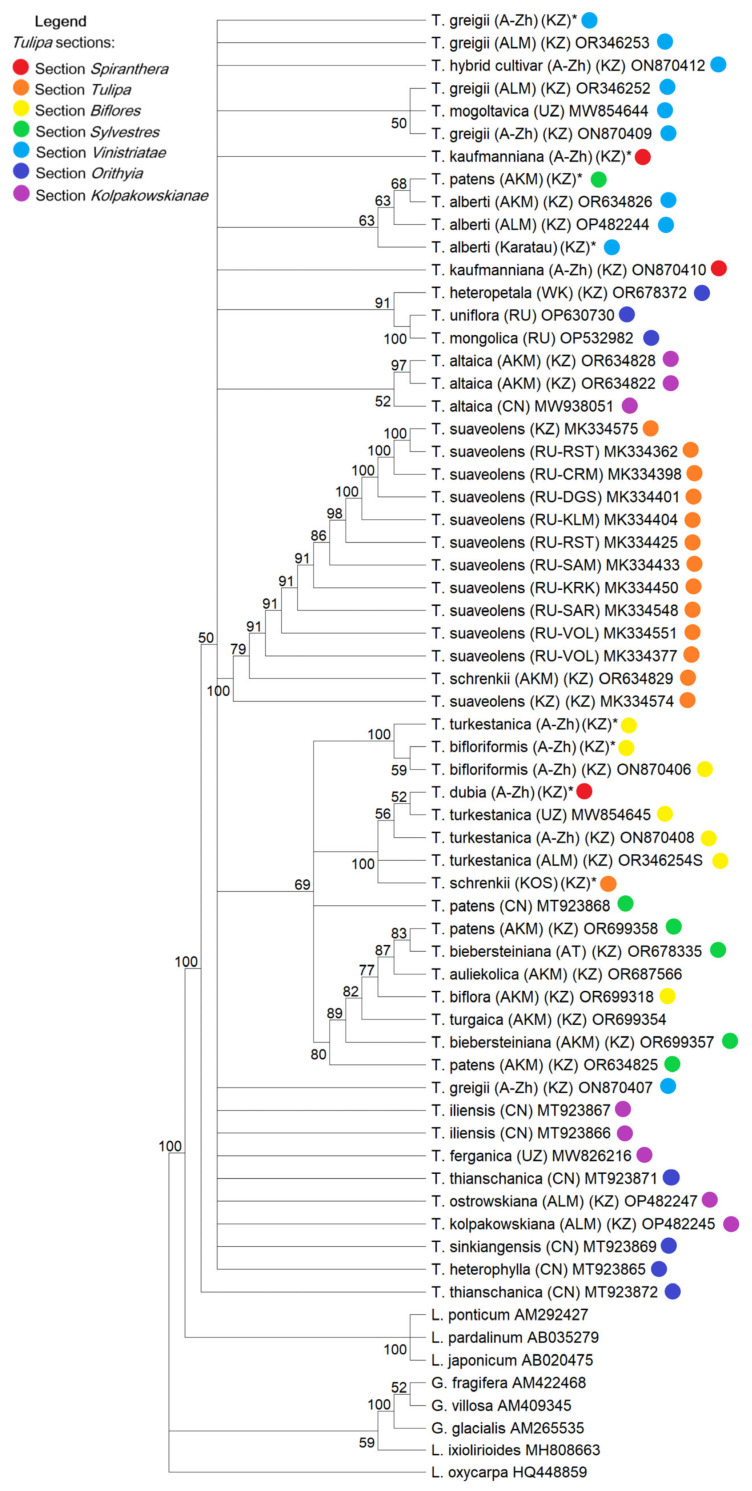
ML phylogenetic tree based on a combined ITS sequence set, with bootstrap values (BS > 50%) provided above each branch. The locations of *Tulipa* samples retrieved from the NCBI GenBank are indicated by capital letters representing the following: Kazakhstan (KZ), China (CN), Russia (RU), and Uzbekistan (UZ). Tulip samples investigated in this study are marked with an asterisk (*). *Tulipa* sections are represented by different colors on the tree.

**Figure 4:**
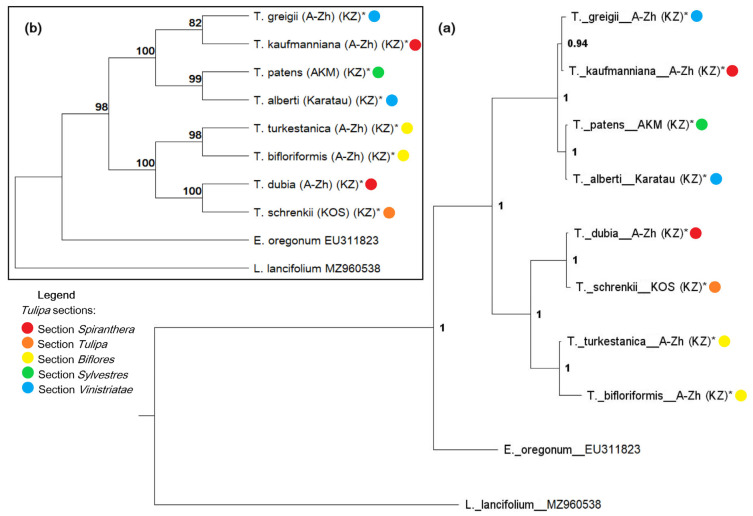
Bayesian inference 50% majority rule consensus tree (**a**) and ML phylogenetic tree (**b**) based on the analysis of the combined *rbc*L + *mat*K + *trn*H-*psb*A + ITS sequence set, including posterior probabilities (PP > 0.5) and bootstrap values (BS > 50%) provided above each branch. Tulip samples investigated in this study are indicated by capital letters representing Kazakhstan (KZ), and marked with an asterisk (*). *Tulipa* sections are represented by different colors on the tree.

The topologies observed in both the BI (Figure 2) and ML (Figure 3) analyses of 65 nrDNA sequence trees from the ITS region were largely congruent, although there were a few unsupported (<0.95 PP, <95% BS) inconsistencies between them. The BI tree revealed four distinct clades, among which a highly supported clade containing representatives of the subgenus *Eriostemones* (0.72 PP, 69% BS) was remarkable. Representatives of the species *T. suaveolens* formed a monophyletic clade with robust support (1.0 PP, 100% BS), and the specimen *T. schrenkii* was assigned to *T. suaveolens*, as these are synonyms (Figure 3 and Figure 4). In contrast to BI, the ML analysis showed lower support and struggled to resolve species within the section *Kolpakowkianae* (Raamsd. ex Zonn. and Veldk.) (0.93 PP, <50% BS) and subgenus *Tulipa* (0.64 PP, <50% BS).

In conjunction with outgroup representatives, the BI tree based on ITS DNA sequences accurately placed *T. alberti* in subgenus *Tulipa* with high statistical support (0.99 PP), whereas *T. patens* (section *Sylvestres* (Baker) Baker) unexpectedly clustered with *T. alberti* (section *Vinistriatae* (Raamsd.) Zonn.) (0.99 PP, 68% BS), complicating the classification of this species (Figure 3 and Figure 4). It is important to note that within the clade of the subgenus *Tulipa*, *T. kaufmanniana* (section *Spiranthera* Vved. ex Zonn. & Veldk.) was placed together with a group of specimens and hybrids of *T. greigii* (section *Vinistriatae* (Raamsd.) Zonn.). There is a hypothesis of natural spontaneous hybridization events between *T. patens* and *T. alberti*, as well as between *T. greigii* and *T. kaufmanniana*, which will be discussed further.

### 2.3. Chloroplast Genome Phylogeny

Overall, the BI (suppl. Figure S1) and ML (suppl. Figure S2) phylogenetic trees generated from the 52 *mat*K region cpDNA sequences of *Tulipa* sp. were similar, and differences between them were not supported. On the BI tree, the section *Kolpakowkianae* had high support (0.81 PP, <50% BS), while the section *Orithyia* (D. Don) Vved. was also well supported (0.98 PP, <50% BS), in contrast to the ML tree. The Kazakh species *T. turkestanica* and *T. bifloriformis* showed a close relationship with the Chinese *T. dasystemon* species (Regel) (section *Biflores* A.D.Hall ex Zonn. & Veldk.) (0.97 PP, <50% BS). Samples of *T. dubia* and *T. schrenkii* were distributed within the clade of the subgenus *Eriostemones* with modest support (0.72 BI, <50% BS). The remaining variants were found scattered throughout the tree within the *Tulipa* clade.

In total, 60 sequences were used to build the BI (suppl. Figure S3) and ML (suppl. Figure S4) phylogenetic trees based on the *rbc*L marker. The species relationships within the genus *Tulipa* showed overall similarity, with some unsupported discrepancies. For instance, the species of the subgenus *Tulipa* formed a single common clade with low support (0.6 PP, 64% BS). Interestingly, according to the ML tree (suppl. Figure S4), species such as *T. dubia* and *T. schrenkii* were grouped together with *T. turkestanica* and *T. bifloriformis*, a grouping not supported by the BI analysis (<0.5 PP, 68% BS) (suppl. Figure S3). All other species were distributed throughout the *Tulipa* clade in the tree.

### 2.4. Combined, rbcL, psbA-trnH, matK, and ITS Data Set of 8 Species of Kazakhstan Tulips

The BI and ML trees, constructed from a concatenated data set of eight sequences of nuclear (ITS) and chloroplast (*rbc*L, *psb*A-*trn*H, and *mat*K) DNA markers showed robust congruence with high support values (Figure 4). Due to insufficient data on the *psb*A-*trn*H marker in NCBI, a separate tree was not generated for this marker. However, the psbA-trnH sequences for the eight tulip species investigated were included in the analysis. Both ML and BI trees resolved into two well-supported clades (1.0 PP, 98% BS). In particular, *T. dubia* and *T. schrenkii* were found to be closely related to the subgenus *Eriostemones* (1.0 PP, 100% BS), whereas *T. patens* showed a close relationship with *T. alberti*, allowing us to confidently places it in the subgenus *Tulipa* (1.0 PP, 100% BS). We also observe the close proximity of the taxa *T. greigii* and *T. kaufmanniana* with high support (0.94 PP, 82% BS).

## 3. Discussion

This study presents a molecular analysis of the genus *Tulipa*, covering a wide range of species, including all available variants from the border regions of Central Asia (such as China, Russia, and Uzbekistan), previously discussed in the literature [5,32,33,37]. These findings complement the existing knowledge of the phylogenetic relationships among species and allow for a more in-depth analysis of their classification. However, some discrepancies between nrDNA- and cpDNA-based phylogenies, especially regarding the placement of certain taxa (*T. patens* and *T. kaufmanniana*), require special attention and discussion.

### 3.1. Incongruent Placement of T. patens in nrDNA and cpDNA Phylogenies

*T. patens* C. Agarth ex Schult. belongs to section *Sylvestres* (Baker) Baker., but the placement of *T. patens* is incongruent with modern tulip taxonomy and differs significantly in nrDNA and cpDNA phylogenies (Figure 3 and Figure 4; suppl. Figures S1–S4). The reasons for this incongruence may be biological (e.g., due to incomplete lineage sorting or introgression between taxa), as commonly observed in many plant groups [38], or due to the conflation of different paralogs in the analysis [39,40].

Conflation of different paralogs seems unlikely, as we thoroughly examined the sequence reads and assemblies and found no evidence of different rDNA paralogs in the samples. On the other hand, introgression, which can have different consequences for nrDNA and cpDNA sequences [41], could potentially have led to the emergence of distinct sequences combining features of different parental lineages. Although putative hybrids between *T. patens* and *T. alberti* have not been documented, *T. patens* often grows in close proximity to *T. alberti* populations in Kazakhstan, making introgression highly likely [8]. Natural hybridization plays an important role in shaping plant diversity, and understanding the mechanisms behind it is crucial for elucidating the evolutionary dynamics of *Tulipa*. Extensive vegetative propagation, often facilitated by factors such as clonal growth or rhizomatous spread, can promote genetic mixing between closely related *Tulipa* species inhabiting overlapping habitats. This process increases the likelihood of hybridization events, leading to the formation of novel genotypes with potentially beneficial traits.

For a more comprehensive understanding of the relationships between *T. patens*, *T. alberti*, and other species in the genus *Tulipa*, broader population sampling and additional nuclear DNA data, preferably from a wide range of loci, are needed. These data can confirm or refute the relationships identified here using nrDNA and cpDNA and can be used to test for evidence of introgression.

### 3.2. Placement of T. kaufmanniana in Section Vinistriatae (Raamsd.) Zonn

As is well known, *T. alberti* Regel and *T. greigii* Regel are representatives of section *Vinistriatae* (Raamsd.) Zonn. [2]. This is confirmed in both the cpDNA and nrDNA trees (Figure 3 and Figure 4, suppl. Figures S1–S4). However, we observe the emergence of *T. kaufmanniana* (section *Spiranthera* Vved. ex Zonn. & Veldk.) within this clade and a closer relationship to it with *T. greigii* in most of the trees generated, including the combined dataset. It is known that *T. greigii* itself is known to be difficult to cultivate due to its susceptibility to the *Fusarium* fungus. Crossbreeding between *T. greigii*, *T. kaufmanniana*, and *T. alberti* is quite common, so hybrids are common both in cultivated areas and in the wild. Numerous hybrids of *T. kaufmanniana* and *T. greigii* have also been reported from the Karshan-tau Mountains [2]. In cases of hybridization between closely related species such as *T. kaufmanniana* and *T. greigii*, which have very similar morphological characteristics, plant identification based solely on morphology alone can be challenging. In such situations, the advantages of DNA barcoding are obvious: it provides a reliable method for species identification, improves taxonomic resolution, and facilitates ecological and evolutionary studies by revealing genetic relationships and ecological interactions.

Although there is uncertainty about the exact placement of *T. kaufmanniana*, as the nodes at the base of the clade are weakly supported in the nrDNA tree, its nested position in the *rbc*L tree and the combined dataset is strongly supported. Considering this, the proximity of *T. kaufmanniana* to *T. greigii*, especially in the cpDNA phylogeny, can be explained by introgression leading to chloroplast capture. Thus, further sampling of *T. kaufmanniana* and additional nuclear DNA markers are needed to assess whether or not there is any incongruence that exists between nuclear and chloroplast DNA relationships and to provide evidence for introgression between the species.

### 3.3. Using a Combined Data Set to Optimize Phylogenetic Analysis

In general, our research demonstrates that the use of individual genetic markers as species-specific barcodes is a convenient and effective approach in molecular genetics. An essential aspect entails the selection of a suitable molecular marker distinguished by substantial variability. This is critical because the degree of variability within a given DNA locus affects the accuracy of phylogenetic analysis and can vary between species. Our studies further confirm the robust reproducibility of the amplification and sequencing results across all DNA markers used. When comparing the DNA sequences of the studied species with well-characterized sequences in the BLAST database, we found a similarity of no less than 98.79%.

When analyzing data related to single markers for phylogenetic tree construction, the use of nuclear ITS sequences yielded higher support values in terms of PP and BS compared with trees constructed from single-plastid markers (*rbc*L and *mat*K) (Figure 3 and Figure 4, suppl. Figures S1–S4) [32,33]. This phenomenon is attributed to the variable nature of the ITS region, which is species-specific and can vary even among closely related organisms, making it widely used in phylogenetic analyses [32,41,42]. It is well known that nrDNA, unlike chloroplast and mitochondrial genomes, accumulates nucleotide substitutions at approximately the same rate [43]. This feature makes the ITS region genetically diverse, allowing the identification of samples not only at the intragenic level but also, in certain cases, at the intraspecific level of variability [32,44,45].

For the more accurate identification and structuring of evolutionary relationships among closely related species, we used a combined dataset that merged information from nuclear and chloroplast DNA (Figure 4). The application of this combined dataset, including sequences of ITS, *rbc*L, *mat*K, and *trn*H-*psb*A, for eight tulip species from Kazakhstan, allowed us to achieve a higher level of resolution and support (PP and BS) in two independent analyses (BI and ML) compared with that in previous studies on Central Asian tulips [5,32,33,37]. The results of the molecular phylogenetic analysis suggest the existence of a previously undescribed hybrid between *T. patens* and *T. alberti*. Furthermore, it is plausible that we have confirmed the presence of a hybrid between *T. kaufmanniana* and *T. greigii* within the territory of Aksu-Zhabagly National Park, as described by Sarsen et al. [32]. However, further detailed population studies are needed to validate this hypothesis. In our study, we not only identified new tulip hybrids, but also confirmed the importance of genetic analysis in developing effective management and conservation strategies for rare and endangered species. Our results have direct practical implications, as they can contribute to the development of new methods for monitoring and controlling the movement and distribution of tulips, as well as helping to conserve their biodiversity. Based on our SNP results, PCR-based tests can be developed to identify tulip species. The discovery of new hybrids also contributes significantly to our understanding of genetic diversity and evolutionary processes in this field.

## 4. Materials and Methods

### 4.1. Sample Collection and Data Acquisition

**Figure 5:**
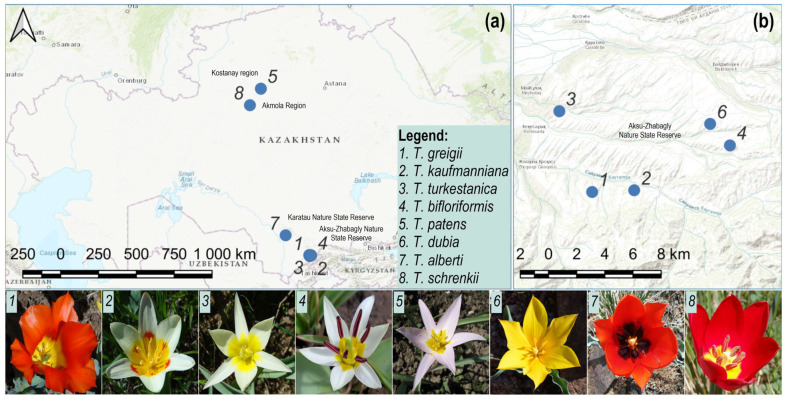
Geographical distribution of *Tulipa* sp. and floral color variability in Kazakhstan. (**a**) Distribution of *Tulipa* sp. throughout Kazakhstan; (**b**) distribution of *Tulipa* sp. within the Aksu-Zhabagly State Nature Reserve. The lower panel presents the range of flower color variation observed in tulips.

All plant material was collected from Aksu-Zhabagly, Karatau Nature State Reserves, and the Kostanay region under the guidance of State Reserve botanists (Figure 5). To address potential sampling errors, our methodology implemented a deliberate sampling strategy by collecting three individuals per species. This approach aimed to provide a robust representation of genetic variability within each species. By including multiple biological replicates of each species, we sought to minimize the risk of misidentification or incomplete representation of genetic diversity. Plant material was identified by the State Reserve botanists using a special identification key from the botanical database [46]. Permission to collect endangered species was obtained from the Forestry and Wildlife Committee of the Ministry of Ecology, Geology and Natural Resources of the Republic of Kazakhstan. The detailed list of accessions is presented in Table 1.

Young flowering plants between 1.5 and 3 months of age were selected for this study to ensure consistency in their physiological state. The corresponding voucher specimens are deposited in a herbarium of the National Center for Biotechnology (Astana, Kazakhstan). To expand the representation of and genetic variation in Kazakh tulip species, data of 154 accessions were downloaded from NCBI GenBank. Outgroup sequences *Paris vietnamensis* (Takht.), *Paris fargesii* (Franch.), *Paris polyphylla* (Sm.), *Gagea lutea* (L.), *Gagea villosa* (M.Bieb.), *Gagea reticulate* (Pall.), *Lilium lancifolium* (Thunb.), *Lilium japonicum f. nobilissimum* (Makino), *Lloydia ixiolirioides* (Baker ex Oliv.), and *Lloydia oxycarpa* (Franch.) taken from GenBank are presented in the electronic Supplementary Materials (Supplementary Appendices S1 and S2) [33]. Reference and outgroup samples were selected based on their appropriate length and ease of alignment.

### 4.2. DNA Isolation, Amplification, and Sequencing

Young leaves of the accessions were stored at −80 °C until DNA extraction. Genomic DNA was extracted using the CTAB method [47] with slight modifications. The extracted DNA was checked for integrity, homogeneity, and purity via 2% agarose gel electrophoresis, run at 120 V for 30 min. The DNA was stored at −20 °C until it was used in the next step of the experiment. Genomic DNA extraction from the samples was performed using three or more independent replicates. All selected samples had high-quality DNA and reached concentrations above 50 ng/μL.

The selection of universal barcode primers was guided by the relevant literature, with comprehensive details provided in Table 4. All primers were synthesized by the Organic Synthesis Laboratory of the National Center for Biotechnology (Astana, Kazakhstan). PCR was performed in a total reaction volume of 40 µL consisting of 2 μL of genomic DNA (50 ng), 0.4 μL of 10× Taq polymerase (Gen Lab, Widnes, UK), 4 µL of 25 mM MgCl_2_ (Thermo Scientific, Walthem, MA, USA), 4 μL of 10× Taq buffer (Thermo Scientific, Waltham, MA, USA), 1 μL of 10 mM dNTP (Thermo Scientific), 1 μL of forward and reverse primers (10 μmol/L stock), and 27.6 µL of ddH_2_O. PCR amplification was performed in T100 Thermal Cycler (Bio-Rad, Berkeley, CA, USA) using the following universal PCR program for all DNA regions: 5 min at 95 °C for initial denaturation (one cycle), 30 cycles of 1 min at 95 °C for denaturation, 1 min at the optimal annealing temperature for each primer (50–58 °C; Table 4), and 1 min at 72 °C for elongation. Finally, one cycle of 10 min was run at 72 °C for extension followed by a 4 °C hold.

The resulting PCR products were verified via 2% agarose gel electrophoresis, run for 30 min at 120 V and purified using PureLink Quick Gel Extraction Kit from Invitrogen. Based on experimental data, the primer annealing temperature was set at 50 °C for *mat*K, 58 °C for *rbc*L, 53 °C for *psb*A-*trn*H, and 55 °C for ITS. The purified PCR products were sequenced via Sanger using a 3730xl DNA analyzer (Applied Biosystems, Foster City, CA, USA). The resulting sequences of both forward and reverse primers from each accession were analyzed using Invitrogen ContigExpress software (Vector NTI Advance 11.5) [52], and contigs were assembled to minimize potential reading errors. The assembled sequences were compared with existing DNA sequences using BLASTn [53] from the National Center for Biotechnology Information (NCBI) GenBank (Bethesda, MD, USA) [54].

### 4.3. Data Analyses

Sequences were aligned in MEGA 11 [55] using automatic algorithm selection and default settings. Aligned sequences were reviewed in BioEdit v. 7.2 [56] and manually realigned. Alignments are available in the electronic Supplementary Materials (suppl. Appendices S3–S10). MrModeltest 2.3 [57] was used to perform model testing prior to Bayesian inference (BI) analyses. The best-fitting models for the nrDNA dataset based on the Akaike information criterion (AIC) were as follows: 18S HKY + I + G, ITS1 SYM + G, and ITS2 HKY + G. For the cpDNA data, the best-fitting models based on the AIC were as follows: CDS GTR + I + G. Bayesian inference was performed in MrBayes v. 3.2 [58]. For nuclear DNA alignment, Markov chain Monte Carlo analyses were conducted with four chains for 10 M generations, sampling every 1000 steps, with a burnin of 25% and an unlinked Dirichlet distribution. For the chloroplast DNA alignment, the analysis was run for 2.5 M generations, sampling every 500 steps [59]. The output files were viewed in Tracer to check for convergence [60]. The average standard deviation of branch frequencies was also checked and confirmed to be below 0.01 at the end of the analyses. Branches with values of < 0.95 PP were considered unsupported.

Maximum likelihood (ML) analysis was performed in MEGA 11 using default settings. Model testing was automated in MEGA 11 using Model Test. The best-fit model for the nrDNA data was ITS TN93+G, and that for the cpDNA sequences were CDS *rbc*L K2+G and *mat*K. The best-fit model for the combined data set was T92+G. Branches with values of less than 95% bootstrap support were considered unsupported. Branches that were supported in one analysis (BI or ML) but not in the other (ML or BI) were considered unsupported overall. The resulting trees were visualized in FigTree v. 1.4.4 [60].

## 5. Conclusions

In summary, our research highlights the effectiveness of using single genetic markers as species-specific barcodes in molecular genetics. The selection of a molecular marker with significant variability is crucial for accurate phylogenetic analysis. We demonstrated the reproducibility of the sequencing results. Analysis of individual markers revealed that nuclear ITS sequences provided better support than plastid markers. The variability of the ITS region allows an accurate identification at both intragenic and intraspecific levels. To improve the accuracy of identifying evolutionary relationships among closely related species, we combined nuclear and chloroplast DNA data sets. This approach significantly improved the resolution and power of the phylogenetic analysis, revealing a previously undescribed hybrid, *T. patens × T. alberti*, and identifying a previously characterized hybrid *T. kaufmanniana × T. greigii*. Our findings suggest the need for further population studies to validate these observations.

## Figures and Tables

**Table 1:** Detailed list of voucher information and GenBank accession numbers of the *Tulipa* samples collected in Kazakhstan*.

Species	Sequence ID	Coordinates	Altitude	Source	Collection Date	*rbc*L	*trn*H-*psb*A	*mat*K	ITS
Subg. *Tulipa* *T. greigii*	T. greigii A-Zh KZ*	42.2039 N, 70.2529 E	1830 m.	Aksu-Zhabagly Nature State Reserve	14-May-2021	ON010708 (583 bp)	ON423208 (464 bp)	ON423211 (732 bp)	OP279724 (727 bp)
Subg. *Tulipa*, *T. kaufmanniana*	T. kaufmanniana A-Zh KZ*	42.205 N, 70.289 E	2050 m.	Aksu-Zhabagly Nature State Reserve	14-May-2021	ON186589 (582 bp)	ON423207 (481 bp)	ON952472 (693 bp)	OP279725 (725 bp)
Subg. *Eriostemones* *T. turkestanica*	T. turkestanica A-Zh KZ*	42.2550 N, 70.2247 E	1340 m.	Aksu-Zhabagly Nature State Reserve	14-May-2021	ON186590 (577 bp)	ON423209 (449 bp)	ON952473 (678 bp)	OP279723 (744 bp)
Subg. *Eriostemones* *T. bifloriformis*	T. bifloriformis A-Zh KZ*	42.2334 N, 70.3713 E	1960 m.	Aksu-Zhabagly Nature State Reserve	15-May-2021	ON186591 (582 bp)	ON423210 (492 bp)	ON952474 (598 bp)	OQ733258 (607 bp)
Subg. *Eriostemones* *T. patens*	T. patens AKM KZ*	51.1112 N, 66.4362 E	272 m.	Akmola Region	28-Apr-2021	OP261551 (574 bp)	OQ718219 (520 bp)	OP261547 (865 bp)	OP279727 (725 bp)
Subg. *Tulipa*, *T. dubia*	T. dubia A-Zh KZ*	42.247 N, 70.3543 E	1910 m.	Aksu-Zhabagly Nature State Reserve	18-May-2021	OP261549 (585 bp)	ON983982 (487 bp)	OQ718220 (818 bp)	OQ733267 (681 bp)
Subg. *Tulipa*, *T. alberti*	T. alberti Karatau KZ*	43.3816 N, 68.3746 E	710 m.	Karatau Nature State Reserve	16-May-2022	OP261548 (564 bp)	ON983980 (528 bp)	OQ718218 (861 bp)	OP279728 (715 bp)
Subg. *Tulipa,* *T. schrenkii*	T. schrenkii KOS KZ*	50.2949 N, 65.5801 E	660 m.	Kostanay region	18-Aug-2022	OP261550 (590 bp)	ON983981 (487 bp)	OP261546 (863 bp)	OP279726 (737 bp)

**Table 2:** Aligned sequence features for *rbc*L, *mat*K, and ITS analyses.

Parameters	rbcL	matK	ITS
No. of taxa	60	52	65
Alignment length (bp)	471	564	485
Conserved sites	461	542	397
Variable sites	10	16	80
Parsimony informative sites	5	6	64
Singleton sites	5	10	16
Overall nucleotide divergence (Pi)	0.002	0.007	0.05
G + C contents (%)	44.6	34.0	60.0

**Table 3:** Single-nucleotide polymorphisms of the ITS, *rbc*L, and *mat*K DNA sequences among *Tulipa* sp. (A: Adenine, C: Cytosine, T: Thymine, G: Guanine).

Species	ITS												*rbc*L			*mat*K		
	28	34	53	260	339	350	362	399	405	426	435	441	117	212	255	174	302	314
*T. greigii*	T	C	A	C	T	C	-	G	T	G	T	A	C	G	A	G	A	A
*T. kaufmanniana*	T	T	A	C	T	C	-	G	T	G	T	A	C	G	A	G	A	A
*T. turkestanica*	C	T	G	C	T	A	G	G	T	G	A	G	T	A	A	G	G	A
*T. bifloriformis*	C	T	G	C	C	A	G	G	T	G	A	G	T	A	G	G	G	A
*T. patens*	T	T	A	T	T	C	-	G	T	A	T	A	C	G	A	G	A	A
*T. dubia*	T	T	A	C	T	C	-	A	C	G	T	A	T	A	A	A	A	A
*T. alberti*	T	T	A	T	T	C	-	G	T	A	T	A	C	G	A	G	A	A
*T. schrenkii*	T	T	A	C	T	C	-	A	C	G	T	A	T	A	A	A	A	T

**Table 4:** Nucleotide sequences of PCR primers used for DNA barcoding.

Primer Name	Nucleotide Sequence of Primer (5′-3′)	Barcoding Locus	Tm (°C)
3F_KIMf [48]	CGTACAGTACTTTTGTGTTTACGAG	matK	50
1R_KIMr [48]	ACCCCATTCATCTGGAAATCTTGGTTC	matK	50
rbcLa_F [49]	ATGTCACCAACAAACAGAGACTAAAGC	rbcL	58
rbcLa_R [49]	GTAAAATCAAGTCCACCRCG	rbcL	58
psbA3f [50]	GTTATGCATGGTGGATTCACAATCC	trnH-psbA	53
trnHf_05 [50]	CGCGCATGGTGGATTCACAATCC	trnH-psbA	53
ITS4 [51]	TCCTCCGCTTATTGATATGC	ITS1 and ITS2	55
ITS5 [51]	GGAAGTAAAAGTCGTAACAAG	ITS1 and ITS2	55

## Data Availability

The DNA barcode sequences of 8 taxa of *Tulipa* sp. mentioned in this paper were uploaded to the GenBank of NCBI (https://www.ncbi.nlm.nih.gov/, accessed on 1 April 2024). The raw data of all DNA barcoding sequences are available in NCBI, and 154 accession numbers are listed in Table 1 and in the electronic Supplementary Materials (Supplementary Appendices S1 and S2).

## Supplementary Materials

The following supporting information can be downloaded at https://www.mdpi.com/article/10.3390/biology13060365/s1.
