# Supplementary material for: Phylogenetic Analysis of Rare and Endangered Tulipa Species (Liliaceae) of Kazakhstan Based on Universal Barcoding Markers
Source: Biology (Basel). 2024 May 22;13(6):365. doi: 10.3390/biology13060365 (PMC11200791; doi:10.3390/biology13060365)
Supplement: Supplementary file 1 [file biology-13-00365-s001.zip › biology-3000739-supplementary/Suppl_Appendix_S1_GB_access.pdf]

**Appendix S1.** GenBank accession numbers of *Tulipa* sp. and Outgroup species

| # | Species/Country/DNA-marker | GenBank accession numbers |
|---|---|---|
| **ITS** | | |
| **Kazakhstan (KZ) ITS** | | |
| 1. | *T. heteropetala* | OR678372.1 |
| 2. | *T. turkestanica* | OR346254.1 |
| 3. | *T. greigii* | OR346253.1 |
| 4. | *T. greigii* | OR346252.1 |
| 5. | *T. patens* | OR699358.1 |
| 6. | *T. biebersteiniana* | OR699357.1 |
| 7. | *T. turgaica* | OR699354.1 |
| 8. | *T. biflora* | OR699318.1 |
| 9. | *T. auliekolica* | OR687566.1 |
| 10. | *T. biebersteiniana* | OR678335.1 |
| 11. | *T. hybrid cultivar* | ON870412.1 |
| 12. | *T. kaufmanniana* | ON870410.1 |
| 13. | *T. greigii* | ON870409.1 |
| 14. | *T. turkestanica* | ON870408.1 |
| 15. | *T. greigii* | ON870407.1 |
| 16. | *T. bifloriformis* | ON870406.1 |
| 17. | *T. suaveolens* | MK334575.1 |
| 18. | *T. suaveolens* | MK334574.1 |
| 19. | *T. ostrowskiana* | OP482247.1 |
| 20. | *T. kolpakowskiana* | OP482245.1 |
| 21. | *T. alberti* | OP482244.1 |
| 22. | *T. schrenkii* | OR634829.1 |
| 23. | *T. altaica* | OR634828.1 |
| 24. | *T. alberti* | OR634826.1 |
| 25. | *T. patens* | OR634825.1 |
| 26. | *T. altaica* | OR634822.1 |
| **Uzbekistan (UZ) ITS** | | |
| 27. | *T. ferganica* | MW826216.1 |
| 28. | *T. turkestanica* | MW854645.1 |
| 29. | *T. mogoltavica* | MW854644.1 |
| **Russia (RU) ITS** | | |
| 30. | *T. uniflora* | OP630730.1 |
| 31. | *T. mongolica* | OP532982.1 |
| 32. | *T. suaveolens* | MK334377.1 |
| 33. | *T. suaveolens* | MK334548.1 |
| 34. | *T. suaveolens* | MK334551.1 |
| 35. | *T. suaveolens* | MK334450.1 |
| 36. | *T. suaveolens* | MK334433.1 |
| 37. | *T. suaveolens* | MK334425.1 |
| 38. | *T. suaveolens* | MK334404.1 |
| 39. | *T. suaveolens* | MK334401.1 |
| 40. | *T. suaveolens* | MK334398.1 |
| 41. | *T. suaveolens* | MK334362.1 |

| | | |
|---|---|---|
| **China (CN) ITS** | | |
| 42. | *T. tianschanica* | MT923872.1 |
| 43. | *T. tianschanica* | MT923871.1 |
| 44. | *T. sinkiangensis* | MT923869.1 |
| 45. | *T. patens* | MT923868.1 |
| 46. | *T. iliensis* | MT923867.1 |
| 47. | *T. iliensis* | MT923866.1 |
| 48. | *T. heterophylla* | MT923865.1 |
| 49. | *T. altaica* | MW938051.1 |
| **Outgroup ITS** | | |
| 50. | *Gagea fragifera* | AM422468.1 |
| 51. | *Gagea villosa* | AM409345.1 |
| 52. | *Gagea glacialis* | AM265535.2 |
| 53. | *Lilium artvinense* | AM292427.1 |
| 54. | *Lilium nevadense* | AB035279.1 |
| 55. | *Lilium japonicum* | AB020475.1 |
| 56. | *Lloydia ixiolirioides* | MH808663.1 |
| 57. | *Lloydia oxycarpa* | HQ448859.1 |
| ***mat*K** | | |
| **Kazakhstan (KZ) *mat*K** | | |
| 1. | *T. patens* | OR703775.1 |
| 2. | *T. turgaica* | OR703767.1 |
| 3. | *T. suaveolens* | OR703757.1 |
| 4. | *T. patens* | OR703755.1 |
| 5. | *T. biflora* | OR703752.1 |
| 6. | *T. turgaica* | OR703743.1 |
| 7. | *T. patens* | OR468324.1 |
| 8. | *T. hybrid* | ON982489.1 |
| 9. | *T. kaufmanniana* | ON982487.1 |
| 10. | *T. greigii* | ON982486.1 |
| 11. | *T. turkestanica* | ON982485.1 |
| 12. | *T. greigii* | ON982484.1 |
| 13. | *T. bifloriformis* | ON982483.1 |
| 14. | *T. buhseana* | OR487146.1 |
| 15. | *T. turkestanica* | OR487145.1 |
| 16. | *T. greigii maturase* | OR487144.1 |
| 17. | *T. kolpakowskiana* | OR487143.1 |
| 18. | *T. alberti maturase* | OR487142.1 |
| **Uzbekistan (UZ) *mat*K** | | |
| 19. | *T. ferganica* | MW828755.1 |
| 20. | *T. intermedia* | MW828754.1 |
| 21. | *T. turkestanica* | MW847260.1 |
| 22. | *T. mogoltavica* | MW847259.1 |
| 23. | *T. vvedenskyi* | MW847258.1 |
| 24. | *T. dubia* | MW847257.1 |
| 25. | *T. affinis* | MW847256.1 |
| **China (CN) *mat*K** | | |
| 26. | *T. thianschanica* | MT917277.1 |

| | | |
|---|---|---|
| 27. | *T. thianschanica* | MT917276.1 |
| 28. | *T. patens* | MT929914.1 |
| 29. | *T. iliensis* | MT929913.1 |
| 30. | *T. iliensis* | MT929912.1 |
| 31. | *T. heterophylla* | MT929911.1 |
| 32. | *T. dasystemon* | MT929910.1 |
| 33. | *T. sinkiangensis* | MT946440.1 |
| 34. | *T. sinkiangensis* | MT946439.1 |
| 35. | *T. gesneriana* | KP711898.1 |
| **Outgroup *mat*K** | | |
| 36. | *Paris vietnamensis* | JF954954.1 |
| 37. | *Paris fargesii* | JF954880.1 |
| 38. | *Paris polyphylla* | AB018828.1 |
| 39. | *Gagea lutea* | KM085624.1 |
| 40. | *Gagea villosa* | KM085631.1 |
| 41. | *Gagea reticulata* | KM085626.1 |
| 42. | *Lilium lancifolium* | KM085639.1 |
| 43. | *Lilium japonicum* | AB049514.1 |
| 44. | *Lloydia ixiolirioides* | MN317534.1 |
| 45. | *Lloydia oxycarpa* | KM085648.1 |
| ***rbc*L** | | |
| **Kazakhstan (KZ) *rbc*L** | | |
| 1. | *T. altaica* | OR738161.1 |
| 2. | *T. heteropetala* | OR738157.1 |
| 3. | *T. sylvestris* | OR738140.1 |
| 4. | *T. biebersteiniana* | OR738117.1 |
| 5. | *T. auliekolica* | OR906710.1 |
| 6. | *T. schrenkii* | OR906709.1 |
| 7. | *T. turgaica* | OR906708.1 |
| 8. | *T. schrenkii* | OR906707.1 |
| 9. | *T. biebersteiniana* | OR906706.1 |
| 10. | *T. alberti* | OR906705.1 |
| 11. | *T. alberti* | OR906704.1 |
| 12. | *T. patens* | OR906703.1 |
| 13. | *T. patens* | OR906702.1 |
| 14. | *T. patens* | OR906701.1 |
| 15. | *T. altaica* | OR906700.1 |
| 16. | *T. biflora* | OR906699.1 |
| 17. | *T. patens* | OR906698.1 |
| 18. | *T. patens* | OR685452.1 |
| 19. | *T. heteropetala* | OR685451.1 |
| 20. | *T. altaica* | OR685450.1 |
| 21. | *T. heteropetala* | OR685449.1 |
| 22. | *T. suaveolens* | OR670282.1 |
| 23. | *T. biebersteiniana* | OR670278.1 |
| 24. | *T. patens* | OR670276.1 |
| 25. | *T. turgaica* | OR670256.1 |
| 26. | *T. patens* | OR468323.1 |
| 27. | *T. ostrowskiana* | OR353398.1 |

| | | |
|---|---|---|
| 28. | *T. kolpakowskiana* | OR353397.1 |
| 29. | *T. greigii* | OR353396.1 |
| 30. | *T. buhseana* | OR353395.1 |
| 31. | *T. turkestanica* | OR353394.1 |
| **Uzbekistan (UZ) *rbc*L** | | |
| 32. | *T. ferganica* | MW828759.1 |
| 33. | *T. intermedia* | MW828758.1 |
| 34. | *T. turkestanica* | MW847266.1 |
| 35. | *T. mogoltavica* | MW847265.1 |
| 36. | *T. vvedenskyi* | MW847264.1 |
| 37. | *T. dubia* | MW847263.1 |
| 38. | *T. affinis* | MW847262.1 |
| 39. | *T. bifloriformis* | MW847267.1 |
| 40. | *T. korolkowii* | MW847261.1 |
| **Outgroup *rbc*L** | | |
| 41. | *Alstroemeria patagonica* | AY120362.1 |
| 42. | *Thismia rodwayi* | AY939892.1 |
| 43. | *Paris vietnamensis* | JF942829.1 |
| 44. | *Paris fargesii* | JF942773.1 |
| 45. | *Paris polyphylla* | D28155.1 |
| 46. | *Gagea lutea* | KM085496.1 |
| 47. | *Gagea villosa* | KM085502.1 |
| 48. | *Gagea reticulata* | KM085498.1 |
| 49. | *Lilium lancifolium* | KM085510.1 |
| 50. | *Lilium japonicum* | AB034921.1 |
| 51. | *Lloydia ixiolirioides* | MN185217.1 |
| 52. | *Lloydia oxycarpa* | KM085518.1 |
