# Supplementary material for: Phylogenetic Analysis of Rare and Endangered Tulipa Species (Liliaceae) of Kazakhstan Based on Universal Barcoding Markers
Source: Biology (Basel). 2024 May 22;13(6):365. doi: 10.3390/biology13060365 (PMC11200791; doi:10.3390/biology13060365)
Supplement: Supplementary file 1 [file biology-13-00365-s001.zip › biology-3000739-supplementary/Suppl_Appendix_S2_GB_access.pdf]

Appendix S2. GenBank accession numbers of outgroup species for the combined dataset

**Species/Sequence ID**	**rbcL**	**trnH-psbA**	***matK***	**ITS**
E. oregonum	KX679106	EU311867	KX677389	EU311823
L. lancifolium	MZ969911	KX346970	MZ970142	MZ960538
