# Supplementary material for: Phylogenetic Analysis of Rare and Endangered Tulipa Species (Liliaceae) of Kazakhstan Based on Universal Barcoding Markers
Source: Biology (Basel). 2024 May 22;13(6):365. doi: 10.3390/biology13060365 (PMC11200791; doi:10.3390/biology13060365)
Supplement: Supplementary file 1 [file biology-13-00365-s001.zip › biology-3000739-supplementary/Supplementary_Figure_legends.pdf]

Supplementary figure legends

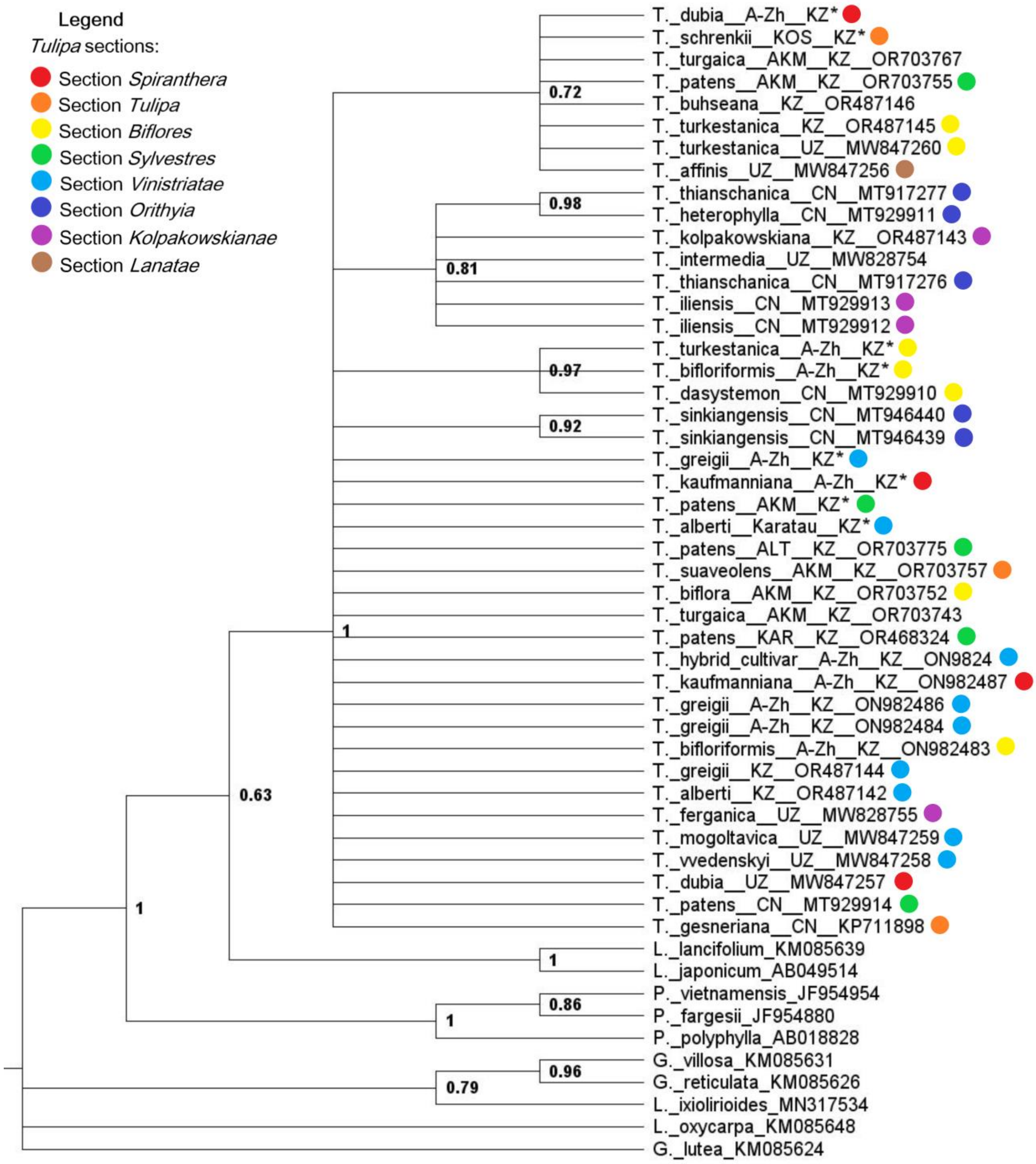

Figure S1. Bayesian inference 50% majority rule consensus tree based on *mat*K sequences, including posterior probabilities (PP > 0.5) provided above each branch. The locations of *Tulipa* samples retrieved from the NCBI GenBank are indicated by capital letters: (KZ) Kazakhstan, (CN) China, (RU) Russia, (UZ) Uzbekistan. Tulip samples investigated in this study are marked with an asterisk (*). Tulipa sections are represented by different colors on the tree.

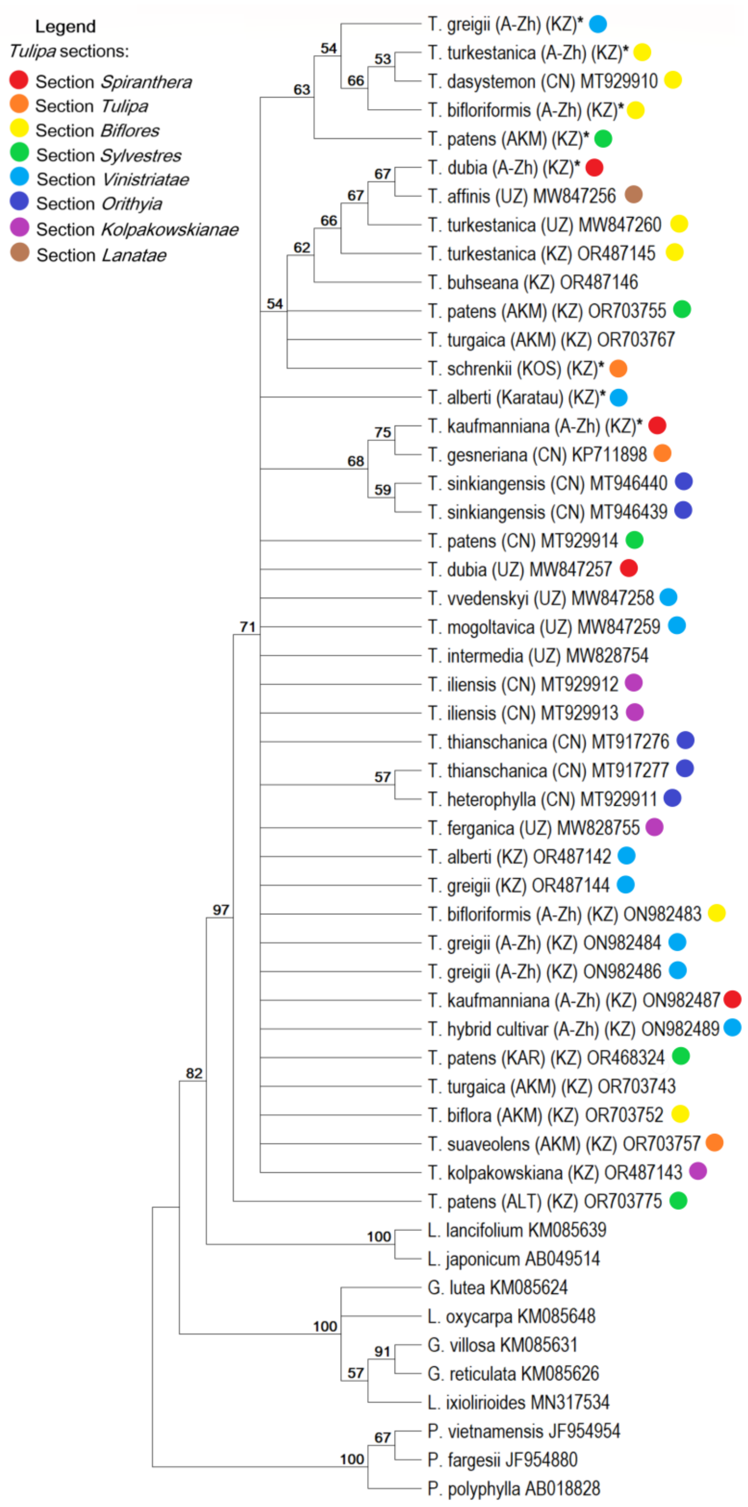

Figure S2. ML phylogenetic tree based on a combined *mat*K sequence set, including bootstrap values (BS > 50%) provided above each branch. The locations of *Tulipa* samples retrieved from the NCBI GenBank are indicated by capital letters: (KZ) Kazakhstan, (CN) China, (RU) Russia, (UZ) Uzbekistan. Tulip samples investigated in this study are marked with an asterisk (*). Tulipa sections are represented by different colors on the tree.

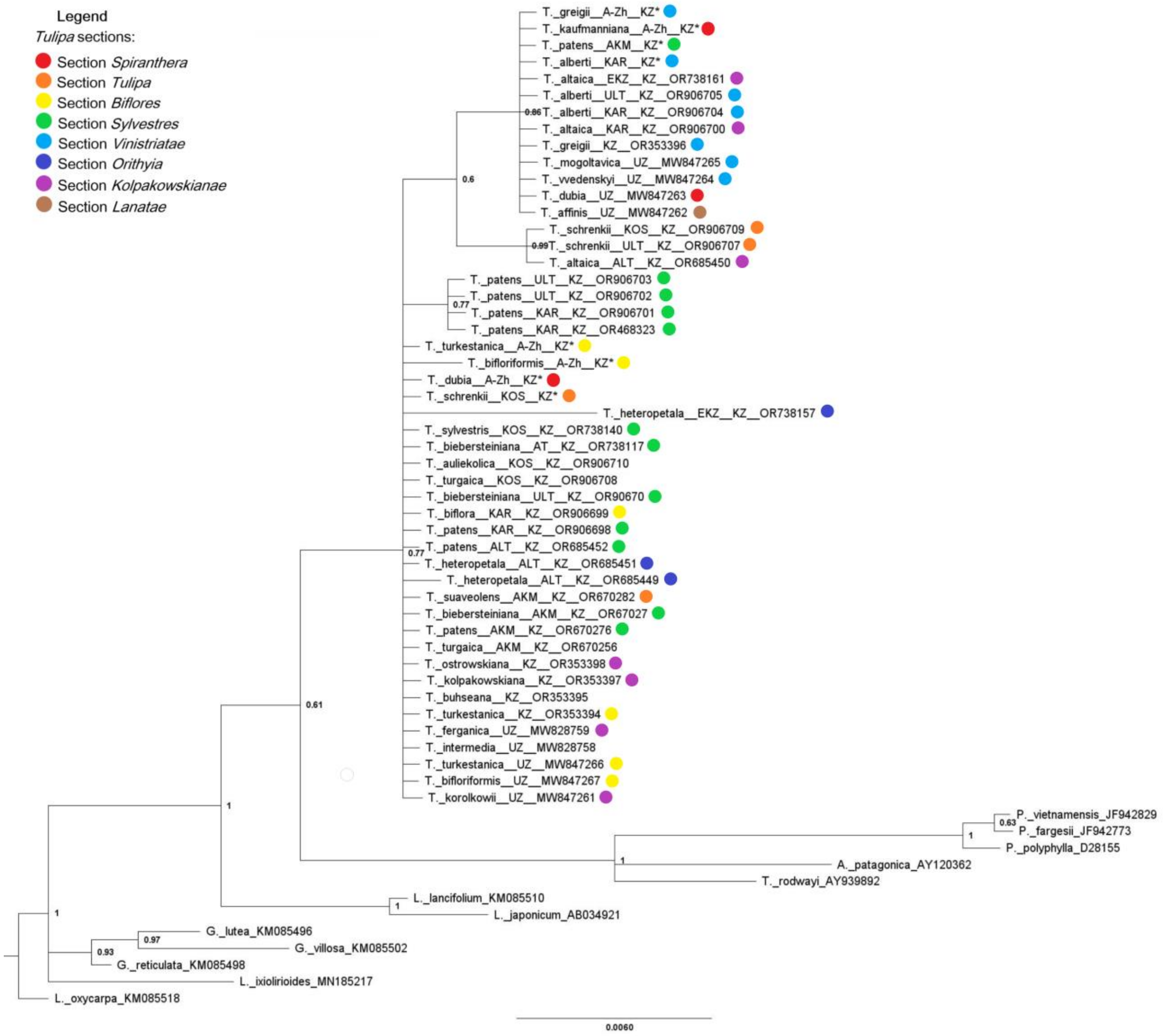

Figure S3. Bayesian inference 50% majority rule consensus tree based on *rbc*L sequences, including posterior probabilities (PP > 0.5) provided above each branch. The locations of *Tulipa* samples retrieved from the NCBI GenBank are indicated by capital letters: (KZ) Kazakhstan, (CN) China, (RU) Russia, (UZ) Uzbekistan. Tulip samples investigated in this study are marked with an asterisk (*). Tulipa sections are represented by different colors on the tree.

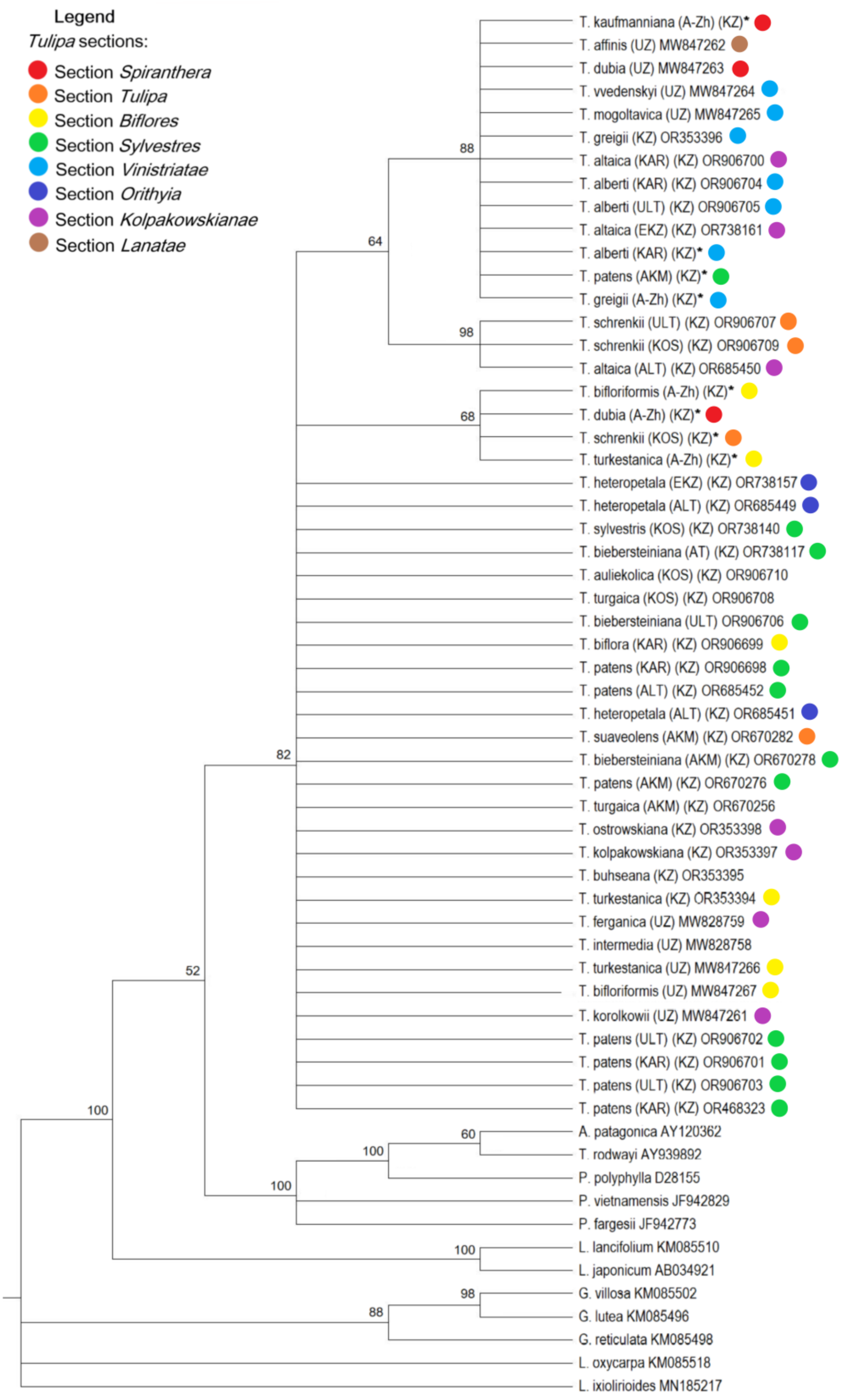

Figure S4. ML phylogenetic tree based on a combined *rbc*L sequence set, including bootstrap values (BS > 50%) provided above each branch. The locations of *Tulipa* samples retrieved from the NCBI GenBank are indicated by capital letters: (KZ) Kazakhstan, (CN) China, (RU) Russia, (UZ) Uzbekistan. Tulip samples investigated in this study are marked with an asterisk (*). Tulipa sections are represented by different colors on the tree.
