# Supplementary figures and images for: Phylogenetic Analysis of Rare and Endangered Tulipa Species (Liliaceae) of Kazakhstan Based on Universal Barcoding Markers
Source: Biology (Basel). 2024 May 22;13(6):365. doi: 10.3390/biology13060365 (PMC11200791; doi:10.3390/biology13060365)

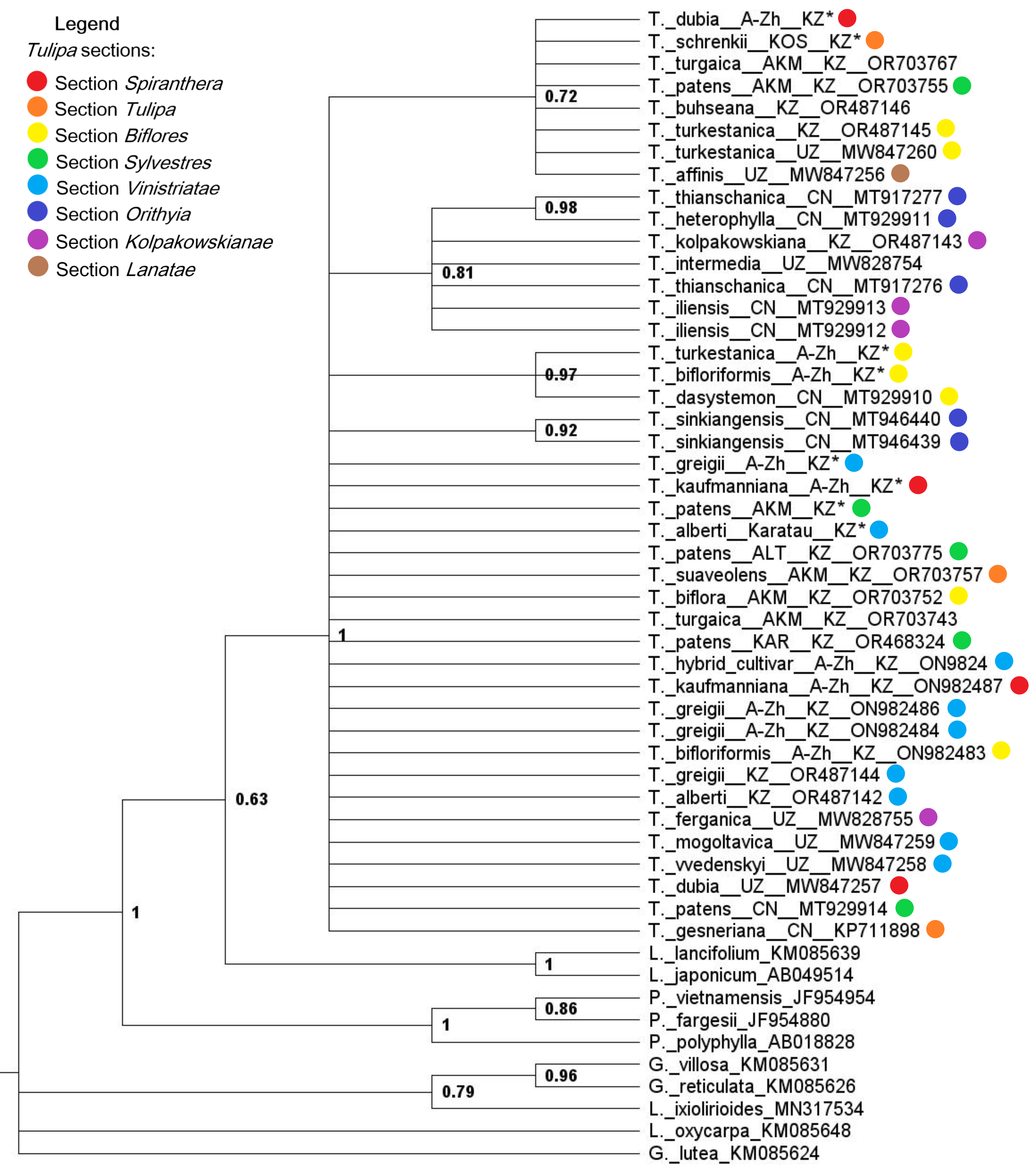

Supplement: Supplementary file 1 [file biology-13-00365-s001.zip › biology-3000739-supplementary/Suppl_Fig_S1_matK_BI_tree.tiff]

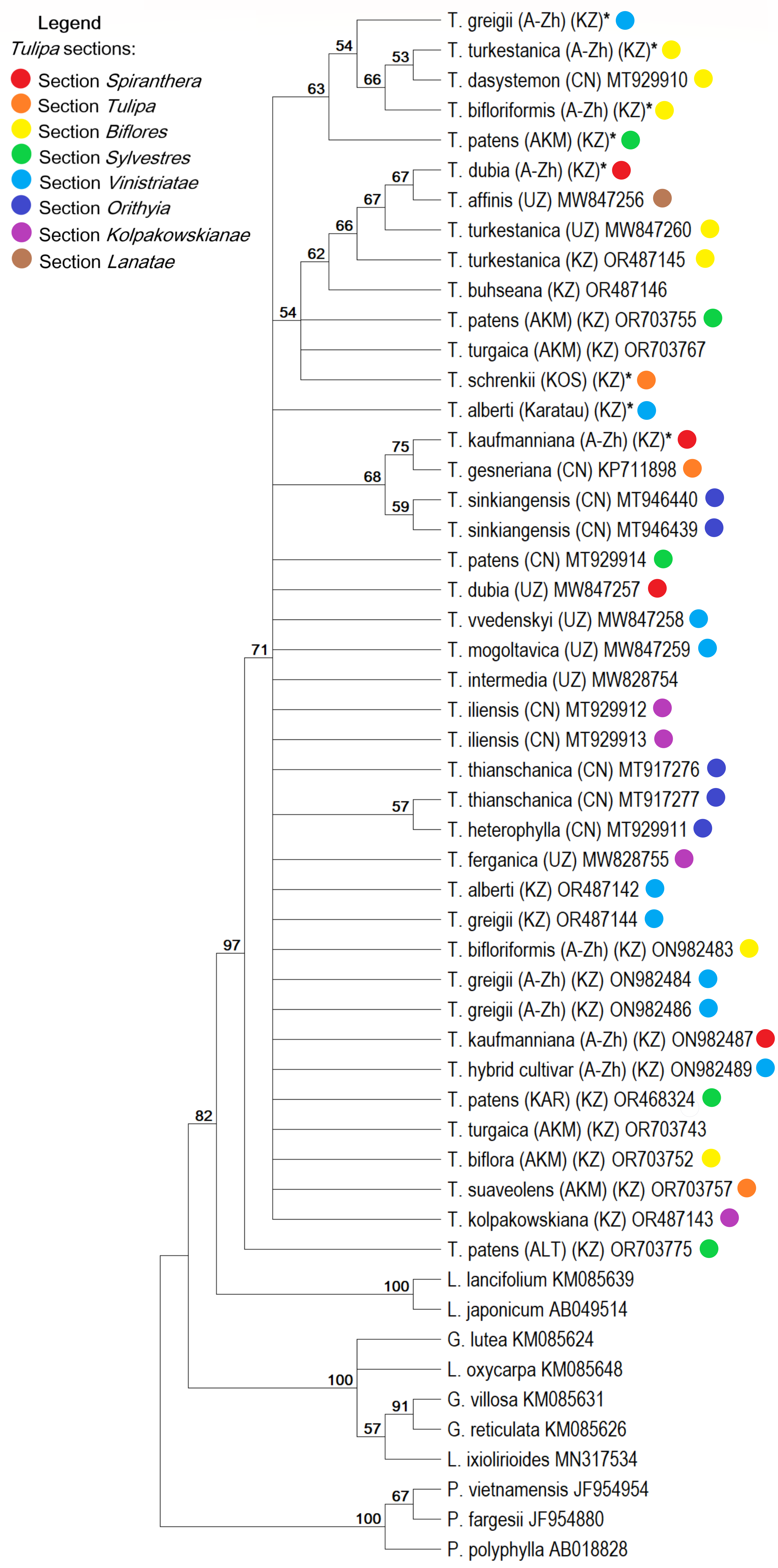

Supplement: Supplementary file 1 [file biology-13-00365-s001.zip › biology-3000739-supplementary/Suppl_Fig_S2_matK_ML_tree.tiff]

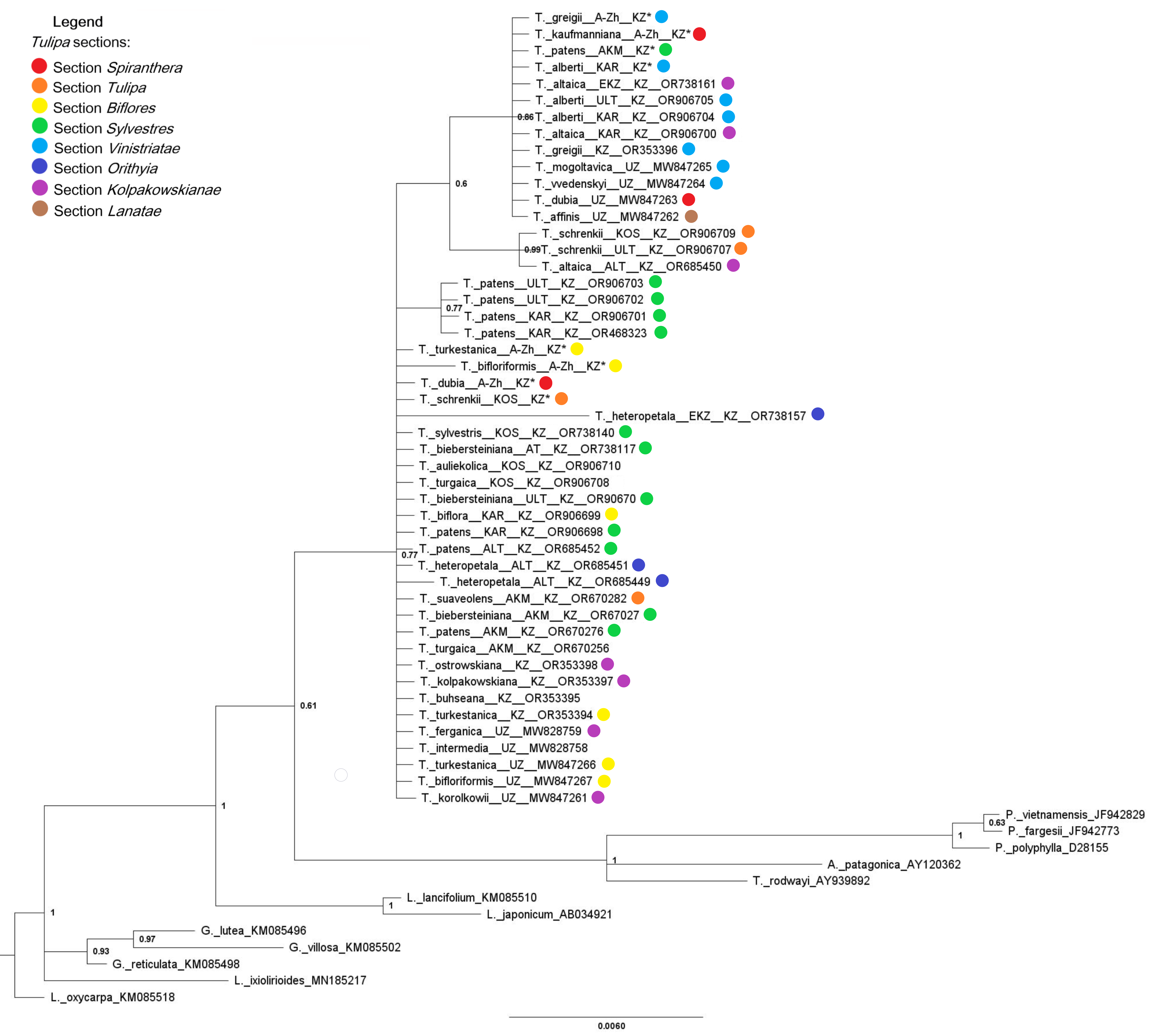

Supplement: Supplementary file 1 [file biology-13-00365-s001.zip › biology-3000739-supplementary/Suppl_Fig_S3_rbcL_BI_tree.tiff]

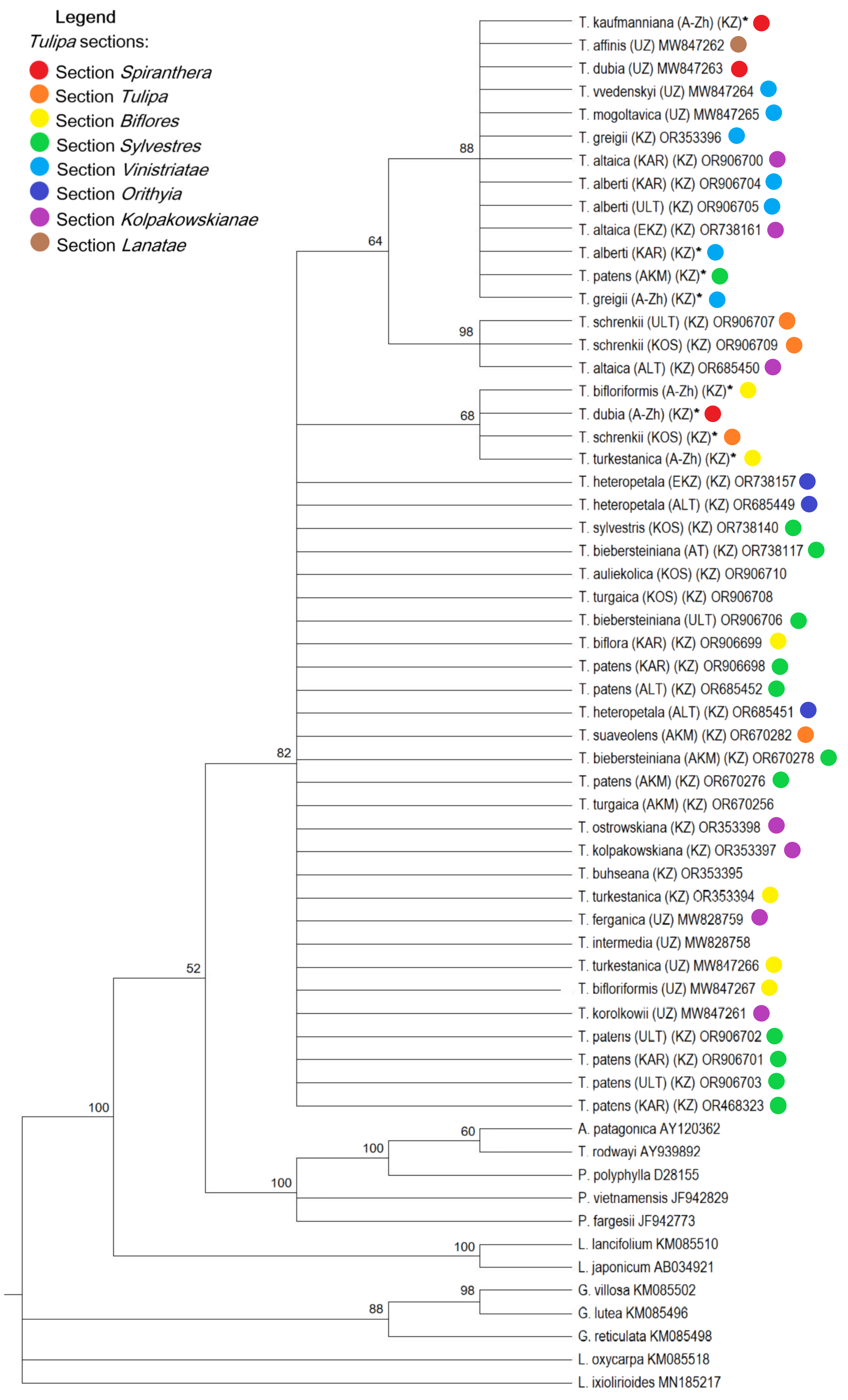

Supplement: Supplementary file 1 [file biology-13-00365-s001.zip › biology-3000739-supplementary/Suppl_Fig_S4_rbcL_ML_tree.tiff]
